# The Impact of Adolescent Resilience on Mobile Phone Addiction During COVID-19 Normalization and Flooding in China: A Chain Mediating

**DOI:** 10.3389/fpsyg.2022.865306

**Published:** 2022-06-23

**Authors:** Anna Ma, Yan Yang, Shuangxi Guo, Xue Li, Shenhua Zhang, Hongjuan Chang

**Affiliations:** ^1^School of Nursing, Xinxiang Medical University, Xinxiang, China; ^2^School of Nursing, St. Paul University Manila, Manila, Philippines; ^3^Department of Neurology, The First Affiliated Hospital of Xinxiang Medical University, Xinxiang, China; ^4^Weihui Senior Middle School, Xinxiang, China

**Keywords:** adolescent resilience, coping style, mobile phone addiction, China, DASS-21, chain mediating, COVID-19, flood

## Abstract

Natural disasters cause long-term psychological problems and increase substance use in some adults. However, it is unclear whether disasters also lead to these problems in adolescents. We hypothesized the influence of adolescent resilience on mobile phone addiction during the normalization of COVID-19 and flooding. We tested the mediating role of coping style and depression, anxiety, and stress (DASS) on phone addiction among 1,751 adolescents in the Henan Province in China. The adolescents were surveyed *via* an online questionnaire, and we used structural equation modeling to examine the correlations and moderation effects. The results show that coping style and DASS could mediate the relationship between adolescent resilience and mobile phone addiction among Chinese adolescents. A chain of coping styles and DASS mediated the relationship between adolescent resilience and mobile phone addiction in Chinese adolescents.

## Introduction

Due to the COVID-19 outbreak, mobile phones have become increasingly important for online teaching and learning in China. According to the 48th China Statistical Report on Internet Development, by June 2021, there were 1.07 billion mobile phone users in China, accounting for 99.7% of the total number of internet users (China Internet Network Information Center, 2021). Furthermore, internet users spend an average of 26.9 h online per week, and the number of internet users aged 6–19 years reached 158 million, accounting for 15.7% of the total percentage of adolescents (China Internet Network Information Center, 2021). As part of normal adolescent psychological development, this age group develops susceptibility to peer influences. In addition, they tend to have low risk perception. These factors increase risk-taking behavior and poor self-regulation (Patton et al., 2016).

Many studies indicated the predictors of mobile phone addiction, such as physiological health (Shoukat, 2019), experiences of childhood abuse (Ma et al., 2020), and self-esteem (Li et al., 2019). Although some studies revealed the relationship between psychological resilience and addiction (Alim et al., 2012; Jebraeili et al., 2019; Shen, 2020; Calpe-López et al., 2022), whether adolescent resilience affects mobile phone addiction remains unclear. Therefore, the present study proposes to test the relationship between adolescent resilience and mobile phone addiction, and the role of coping style and mental health during the normalization of COVID-19 and flooding. The relationships between the variables mentioned above are described in the following sections.

### Psychological Resilience and Mobile Phone Addiction

According to resilience framework theory, psychological resilience is the ability to cope with a crisis or quickly return to a pre-crisis status mentally or emotionally (de Terte and Stephens, 2014). Previous studies found that psychological resilience was related to substance addiction (Alim et al., 2012) or behavioral addiction (Goldstein et al., 2012). In numerous studies, psychological resilience has been shown to predict video game addiction (Turan, 2021) and internet addiction (Zhou et al., 2017). Despite the close relationship between adverse childhood experiences (ACE) and addictive behaviors, researchers have not explored the relationship between psychological resilience and mobile phone addiction. Thus, the possible mechanism of how psychological resilience affects mobile phone addiction needs to be researched further.

### Coping Style and Mobile Phone Addiction

Coping strategies are psychological models used by individuals to manage emotions, thoughts, and behaviors when they encounter various states of stress in different stages, reflecting all responses to stress that can be utilized and successfully used (Carver and Connor-Smith, 2010). Many studies have explored the relationship between coping strategies and behavioral problems (Windle and Windle, 1996; Liu et al., 2004). Gharaei et al. (2008) showed that some changes related to behavioral disorders were explained by coping strategies. They found that restraint coping was negatively associated with Internet addiction (Chou et al., 2015). Based on the relationship between coping strategies and addictive behaviors, the current study assumed that coping styles would predict mobile phone addiction.

### Stress, Anxiety, and Depression, and Mobile Phone Addiction

Stress is an experience of negative emotions and behavioral changes. The environment plays an important role in generating stressors (Baum, 1990). Regarding the emotional state of adolescents, stressful life events, such as natural disasters, have been identified as a significant risk factor. Makwana (2019) indicated that the psychological effects of a disaster were more severe among children, women, and the dependent elderly population. Furthermore, research showed that youth experiencing high levels of exposure to such disasters had the highest mean levels of life stressors (Felix et al., 2020). Jermacane et al. (2018) found that anxiety and depression among survivors of widespread flooding in the United Kingdom had a prevalence of over 10% 2 years post-disaster.

In July 2021, the Henan province suffered from unusually heavy rainfall and maximum continuous rainfall of 958 mm (The Tenth Press Conference of Henan Province Flood Control and Disaster Relief, 2021), causing severe flooding. The flooding, referred to as the “7.20 Henan rainstorm,” quickly destroyed the overwhelmed dams and river banks, causing severe traffic paralysis, water power failure, and waterlogging. It upended tens of millions of lives. The government called on people to stop working and attending school, and actively organized personnel to launch necessary rescue missions. Many adolescents and their parents were trapped in their homes, and some parents volunteered to help with relief efforts. Flooding causes severe stress which continues long after the waters recede (Felix et al., 2020). This prolonged stress can induce behavioral problems in children, lead to grief and economic difficulties for families, increase substance use and misuse, exacerbate existing problems, and negatively impact mental health (Stanke et al., 2012). The present study supposes that stress, anxiety, and depression positively relate to mobile phone addiction.

### Psychological Resilience, Coping Style, and Stress, Anxiety, and Depression

Children who had more exposure to the flood events had greater resilience than those who had less or none (Arshad et al., 2020). Resilience is a dynamic process that significantly contributes to survivors successfully recovering from disasters (Bonanno et al., 2010). Previous studies have also shown that disaster exposure was positively associated with substance use and was negatively related to children’s psychological resilience (Fuchs et al., 2021). Indeed, Bonanno and Galea (2007) found that resilient participants were less likely than others to smoke and use marijuana after a traumatic experience. In addition, they found positive coping and resilience were protective factors for the emergence of stress, anxiety, and depressive symptoms in junior high and high school students (Zhang et al., 2020). Though adolescent mental health was impacted by COVID-19 and its variants during the study period, there were no new cases of COVID-19 in our region (Central People’s Government of the People’s Republic of China, 2022), there was no lockdown, and everyone could enter public places with health Quick Response (QR) codes and masks. Therefore, this study focuses on the impact of stress caused by sudden floods on adolescents’ mental health in normalizing epidemic prevention and control.

Many scholars believe that adolescent resilience is also related to substance use, such as smoking and excessive drinking (alcoholism) (Davis and Spillman, 2011). Some experts also believe that depression, anxiety, and pressure may lead to internet addiction (Carli et al., 2013). However, studies on the relationship between mental resilience and adolescents’ coping styles, mental health, and mobile phone addiction are rare.

Given this, adolescents confined to their homes may be likely to overuse mobile phones and the internet due to floods in China. Thus, we hypothesized a correlation between resilience and mobile phone addiction among adolescents, and that coping style and mental health play a mediating role in that relationship.

### Associations Between Adolescent Resilience, Coping Style, Mental Health, and Mobile Phone Addiction Outcomes

As a stressor, flooding can affect adolescent mental health and cause symptoms of anxiety and depression. Many studies show that resilience eliminates the symptoms of anxiety and depression, and increases self-esteem and mental well-being among young people (Downie et al., 2010; Baldwin et al., 2011). Studies have found that young people with high resilience have fewer mental health problems (Sood et al., 2013). Adolescents with different levels of resilience may have different coping styles when faced with stress. Furthermore, research has shown that adolescents with lower self-esteem engage in the coping strategies of ventilating feelings, avoiding problems, and relaxing. Adolescents with higher self-esteem are more likely to engage in coping styles that directly address their problems (Chapman and Mullis, 1999). The purpose of escapism is to escape from stress and the dilemma of reality. A study found that escapism mediates psychological grief and internet addiction (Ohno, 2016). Problematic mobile phone use was positively associated with mental health problems and escapism (Atış Akyol et al., 2021).

Resilience is a crucial developmental stage during adolescence, as it is a transitional period characterized by significant neurobiological and psychosocial changes in amplifying environmental demands and increasing sensitivity to social contexts (Schriber and Guyer, 2016). During the COVID-19 pandemic, children who used positive strategies to cope with the situation suffered less emotionally and behaviorally (Orgilés et al., 2021). Shao found a significant positive correlation between resilience and positive coping styles among middle school students in China, indicating that adolescents with high psychological resilience are more inclined to adopt a positive coping style (Shao et al., 2021). Psychological resilience is the ability to cope with a crisis or quickly return to a pre-crisis status mentally or emotionally (de Terte and Stephens, 2014). Resilience and positive coping are protective factors for the emergence of stress and anxiety symptoms in adolescents. The mediating role of parents’ stress influences children’s emotional and behavioral problems (Dąbkowska et al., 2021). The mutually enhancing relationship between resilience and positive mental health, and vice versa, a mutually reducing relationship between resilience and mental illness, presented the significant influence of mental health level on resilience (Wu et al., 2020). Malek showed that avoidant coping styles could aggravate depressive, anxiety, and stress symptoms during the COVID-19 pandemic. Conversely, applying resilience and approach-based coping techniques can decrease the mental health burden of the pandemic on participants (Smida et al., 2021). Researchers Brennan (2007), Ali et al. (2010), and Tempski et al. (2015) have indicated that enhancing resilience reduces behavioral problems and the risk of problematic mental health.

**FIGURE 1:**
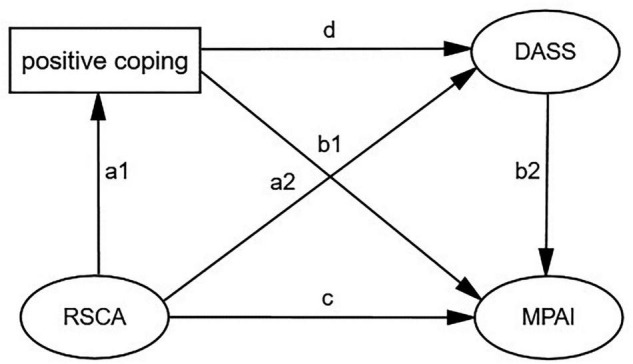
Hypothesized model. MPAI, Mobile Phone Addiction Index; DASS-21, Depression, Anxiety, and Stress Scale with 21 Items; RSCA, the Resilience Scale for Chinese Adolescents; SCSQ, the Simplified Coping Style Questionnaire.

Based on the literature review, the present study constructed a chain mediation model to examine the mediating role of positive coping, stress, anxiety, and depression in the relationship between adolescent resilience and mobile phone addiction among Chinese adolescents. Furthermore, we proposed a model to test the associations among Chinese adolescent resilience, coping style, mental health, and mobile phone addiction to further clarify mobile phone addiction related to resilience (Figure 1).

## Materials and Methods

### Participants

Convenience sampling was employed to recruit students from grades 7–9 in middle school and grades 1–3 in high school in Henan province of China. Inclusion criteria: (1) flood areas in Henan Province; (2) junior or senior high school students, aged 12–18; (3) floods from July to August 2021; and (4) informed consent and voluntary participation in this study. Exclusion criteria: (1) teenagers who are not in school and (2) students who have communication problems. All subjects signed informed consent online before participating in the study, which was authorized by the Human Participant Review Committee of Xinxiang Medical College. All projects are available in an easy-to-understand Chinese version. Trained graduate students in psychology handed out and recycled the scales. They are also responsible for explaining possible doubts to avoid any confusion. It takes about 20 min to fill out the questionnaire. They completed online questionnaires from July 1 to August 30, 2021. A total of 1,751 valid questionnaires were obtained, with an effective response rate of 97.28%.

### Measurement of Structures

#### Depression, Anxiety, and Stress Scale With 21 Items

The Depression, Anxiety, and Stress Scale with 21 Items (DASS-21) was used to evaluate negative emotional states of DASS (Lovibond and Lovibond, 1995), referring to the previous week, with each item classified into four Likert responses from 0 to 3 (0 = *never* to 3 = *most of the time*). This self-report instrument includes three subscales: (1) the stress subscale, which measures tension, agitation, difficulty relaxing, and negative affection; (2) the anxiety subscale, which assesses autonomic arousal, skeletal musculature effects, situational anxiety, and subjective experience of anxious arousal; and (3) the depression subscale, which measures hopelessness, dysphoria, lack of interest, self-deprecation, and inertia. The reliability coefficients of depression, anxiety, and stress were 0.82, 0.82, and 0.79, respectively. The Cronbach’s alpha of the total scale was 0.89.

#### Mobile Phone Addiction Index

Leung designed the Mobile Phone Addiction Index (MPAI) to identify addiction symptoms associated with mobile phone use among adolescents in Hong Kong (Leung, 2008). The scale includes 17 items answered on a five-point Likert scale of 1 to 5 (1 = *not at all*; 2 = *rarely*; 3 = *occasionally*; 4 = *often*; and 5 = *always*). The scale covers four dimensions: (1) “inability to control craving,” which reflects the amount of time adolescents spend on their mobile phones, thereby leading to complaints from family and friends about their compulsive mobile phone use and causing the adolescents to lose of sleep due to excessive use; (2) “anxiety and feeling lost” assesses preoccupation, feeling lost or anxious, and having difficulty switching off the mobile phone; (3) “productivity loss” measures decreased productivity and diverted attention from pressing issues due to adolescents’ excessive use mobile phones; and (4) “withdrawal and escape” indicates that adolescents use their mobile phones to escape from isolation, loneliness, and feeling down. The Cronbach’s alpha of the scale was 0.90.

#### Resilience Scale for Chinese Adolescents

The Resilience Scale for Chinese Adolescents (RSCA) was developed by Hu and Gan (2008) according to the process model of the resilience concept and applied to Chinese adolescents. There are 27 items divided into two factors: “manpower” and “support.” The former includes three factors: goal focus, emotion control, and positive cognition. The latter consists of two factors: family support and interpersonal assistance. The reliability of the total scale was 0.85.

#### Simplified Coping Style Questionnaire

This instrument was designed by Xie (1998) to simplify and modify non-Chinese coping style scales. A simple coping style questionnaire constructed from the perspective of Chinese cultural characteristics, it is composed of 20 items concerning strategies and attitudes people may adopt in everyday life to confront setbacks. The coping styles are divided into two categories: “positive coping” and “negative coping.” The reliability of the total scale was 0.90, while the positive coping and negative response subscales were 0.89 and 0.78, respectively.

### Data Analysis

All data analyses were performed using SPSS 26.0 and Amos 23 (IBM Inc., Armonk, NY, United States). First, descriptive data were received using SPSS 26.0, and correlations variables were calculated using Pearson’s correlations. Second, according to Baron and Kenny (1986), we analyzed the mediation effects using two measurement models to examine how well the indicators represented each latent variable. Second, we tested the hypothesized relationships among latent variables. Maximum likelihood (ML) estimation was used to test the two structural models in the AMOS 23.0 program. When Tucker-Lewis index (TLI) >0.90, comparative fit index (CFI) >0.90, and Root Mean Square Error of Approximation (RMSEA) <0.06, the model fits well, according to Hu and Bentler (1999). We followed the stepwise method to structure the best-fitting model for the mediated effects and bootstrapping with 5,000 replications to measure the chain mediation model. All data analyses were two-tailed, with significance levels of *P* < 0.01 and *P* < 0.05.

## Results

### Descriptive Statistics

The demographic profiles and descriptive statistics for the final analysis of the participants are provided in Table 1. We included 1,751 participants. There were 727 (41.519%) participants in middle school; 1,024 (58.480%) in high school; 142 (8.110%) from single-child families; and 1,609 (91.890%) participants from non-single-child families.

**TABLE 1:** Demographic profiles and descriptive statistics of the participants.

Variables	Frequency	Percentage
Gender		
Boy	725	41.405
Girl	1,026	58.595
Single child		
yes	142	8.110
no	1,609	91.890
Birth order		
1st	854	48.772
2nd	802	45.802
3rd	95	5.425
Nationality		
Han	1,743	99.543
Hui	7	0.400
Miao	1	0.057
Grade		
Middle school (7th)	155	8.852
Middle school (8th)	568	32.439
Middle school (9th)	4	0.228
High school 1st	34	1.942
High school 2nd	471	26.899
High school 3rd	519	29.640
Total	1,751	100.0

### Univariate Analysis and Correlation Analysis of Major Study Variables

As displayed in Table 2, for the 1,751 participants’ results, the category total means (SD) are as follows: MPAI, 7.954 (±3.987); DASS-21, 5.190 (±4.566); positive coping, 10.588 (±5.123); negative coping, 16.068 (±4.741); RSCA, 39.833 (±13.555).The variables correlated with the constructs in Table 3 were less than 0.85. The discriminant validity value (<0.85) was met in the construct correlation (Kline, 2005). These findings showed that valid and reliable constructs were used.

**TABLE 2:** Basic characteristics and measure scores.

	*M*	SEM	Frequency	Percentage
Age	15.165	2.352		
MPAI total	7.954	3.987		
Feeling anxious and lost	16.808	5.797		
Inability to control craving	7.758	3.271		
Productivity loss	7.314	3.456		
Withdrawal	13.609	12.674		
DASS-21 total	5.190	4.566		
Stress	4.060	4.234		
Normal			1,669.000	95.317
Mild			58.000	3.312
Moderate			24.000	1.371
Severe			0.000	0.000
Extremely severe			0.000	0.000
Anxiety	4.359	4.666		
Normal			1,463.000	83.552
Mild			95.000	5.425
Moderate			138.000	7.881
Severe			39.000	2.227
Extremely severe			16.000	0.914
Depression	19.798	7.497		
Normal			1,519.000	86.750
Mild			120.000	6.853
Moderate			92.000	5.254
Severe			20.000	1.142
Extremely severe			0.000	0.000
SCSQ total	58.908	18.792		
Positive coping	10.588	5.123		
Negative coping	16.068	4.741		
RSCA total	39.833	13.555		
Focused	18.829	5.759		
Interpersonal support	20.111	5.538		
Emotional control	14.375	3.659		
Positive cognitive	16.974	4.417		
Family support	89.967	17.790		

**TABLE 3:** Correlation analysis of study variables.

	1	2	3	4
1. RSCA total	–			
2. DASS-21 total	−0.661**	–		
3. MPAI total	−0.413**	0.561**	–	
4. Positive coping	−0.323**	0.470**	0.354**	–

**P < 0.01.

### Structural Model Testing and Structural Relationship Between Constructs

**FIGURE 2:**
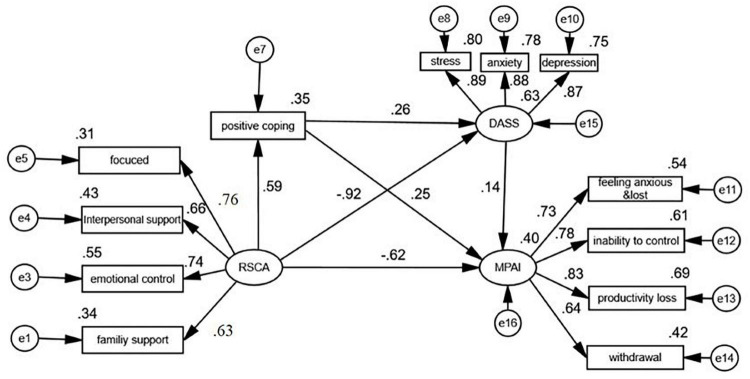
The standardized path coefficients in model testing. MPAI, Mobile Phone Addiction Index; DASS-21, Depression, Anxiety, and Stress Scale with 21 Items; RSCA, the Resilience Scale for Chinese Adolescents; SCSQ, the Simplified Coping Style Questionnaire.

The test results revealed the goodness of fit of the proposed structural model (χ^2^/df = 2.85, RMSEA = 0.047, goodness-of-fit index (GFI) = 0.982, CFI = 0.987). The hypothesis relationships between the variates are demonstrated in Table 4 and Figure 2. The indirect effects are presented in Table 5. Bootstrapping analyses (5,000 process repetitions) showed that the indirect effects of adolescent resilience on mobile phone addiction through positive coping and stress, anxiety, and depression were significant and positive (standardized indirect effect 0.112, 95% CI [0.057, 0.179], *P* < 0.01). The indirect effect of adolescent resilience on mobile phone addiction through stress, anxiety, and depression was −0.602, 95% CI [−0.88, −0.298], *P* < 0.01, excluding 0. The mediating effect was significant. The indirect effect of adolescent resilience on mobile phone addiction through positive coping was 0.122, 95% CI [0.038, 0.231], *P* < 0.01, excluding 0, and the mediating effect was significant. In Figure 2, the factor loading of the “positive cognitive” variable of RSCA is less than 0.5, which indicates that the reliability of the observed variable is not good and cannot reflect the true meaning of the latent variable to a certain extent, so it was deleted.

**TABLE 4:** Results of the structural model: tests of hypothesized associations between constructs.

	Estimate	SE	*t*-Value	*P*
Positive coping ← RSCA	1.909	0.096	19.801	***
DASS ← RSCA	−1.723	0.088	−19.514	***
DASS ← positive coping	0.217	0.019	11.665	***
MPAI ← DASS	0.318	0.049	6.505	***
MPAI ← RSCA	−0.328	0.108	−3.042	0.002
MPAI ← positive coping	0.070	0.019	3.725	***

***P < 0.001.

**TABLE 5:** Bootstrap truncated regression results.

				Bootstrapping				
				
		Product of coefficients		BC 95% CI		Percentile 95% CI		
				
Relationships	Point estimate	SE	*Z*	Lower	Upper	Lower	Upper	*P*
**Indirect effects**								
RSCA → positive coping → MPAI	0.122	0.048	2.542	0.042	0.234	0.038	0.231	0.003
RSCA → DASS → MPAI	−0.602	0.15	−4.013	−0.889	−0.299	−0.888	−0.298	0.002
RSCA → positive coping → DASS→ MPAI	0.112	0.032	3.500	0.061	0.186	0.057	0.179	0.002
Total	−0.737	0.053	−13.906	−0.847	−0.639	−0.847	−0.637	0

## Discussion

### Direct Relations

Mobile phones are regarded as a necessity of modern life. With the increasing incidence of mobile phone addiction among adolescents, many researchers are focusing on potential risk factors leading to mobile phone addiction. To date, there is little research on the relationship between psychological resilience and mobile phone addiction in adolescents. To address this gap, this study surveyed how adolescent resilience, coping style, DASS affect mobile phone addiction among Chinese adolescents. The results showed that adolescent resilience could directly and negatively affect mobile phone addiction in Chinese adolescents. In other words, adolescents with lower levels of psychological resilience show an increased propensity for mobile phone addiction. This result is consistent with previous research findings (Robertson et al., 2018). According to the resilience framework theory (Kumpfe and Bluth, 2004), psychological resilience is an important protective factor for problem behavior and personal mental health. Griffiths (2005) argued that addictions consist of several components, such as relapse, mood modification, tolerance, conflict, and withdrawal. The findings of Shen (2020) demonstrated that psychological resilience is correlated with excessive smartphone use. However, they did not reveal whether the correlation was positive or negative. A study by Yaqiong et al. (2017) revealed that resilience negatively predicted mobile phone addiction. This result was consistent with other research findings. Such findings suggest that we could enhance psychological resilience levels to reduce the risk of mobile phone addiction.

### Mediated Role

Positive coping styles and DASS play intermediary roles in adolescent psychological resilience and mobile phone addiction in Chinese adolescents, respectively; thus, our hypothesis was supported. This is consistent with previous studies. Understanding and managing coping styles can be particularly effective for addressing smartphone addiction (Alan and Guzel, 2020). The present study found that a positive coping style could moderate the relationship between adolescent psychological resilience and mobile phone addiction. Specifically, the indirect effect of psychological resilience on mobile phone addiction is moderated and buffered by a positive coping style. This result suggests that a positive coping style could help improve psychological resilience levels. Improved psychological resilience will reduce mobile phone addiction risk. The Simplified Coping Style Questionnaire (SCSQ) revealed that coping style had a robust effect on adolescent mobile phone addiction (Lu et al., 2021).

Depression, anxiety, and stress could moderate the relationship between adolescent psychological resilience and mobile phone addiction. Adverse COVID-19 experiences and exposure to flooding can lead to social isolation and unmet basic psychological needs, resulting in adolescent anxiety, depression, and other unpleasant or pathological psychological states. The online environment or use of a mobile phone could provide a temporary escape from unpleasant experiences and stress in the real world. However, using a mobile phone compulsively to acquire satisfaction and happiness may eventually result in addiction. Many studies have indicated a relationship between smartphone use and depression, anxiety, and loneliness (Elhai et al., 2018; Kim et al., 2019). Depression and social anxiety are risk factors for more problematic smartphone use (Pera, 2020). Stress, anxiety, and depression were significantly positively correlated with smartphone addiction (Choksi and Patel, 2021). Researchers found a significant positive relationship between anxiety about COVID-19 infection and the number of daily smartphone use hours. The strongest predictor of smartphone addiction was anxiety about COVID-19 infection (Al Qudah et al., 2021).

### The Chain Mediating Role

Positive coping style and DASS played a continuous intermediary role in the impact of adolescent resilience to mobile phone addiction among Chinese adolescents. Smartphone users who experience depressive symptoms may similarly use their mobile devices as a coping strategy to alleviate these (Ahn and Kim, 2015). Coping and affective disorders play critical roles in international addiction among adolescents (Einar, 2017). Stressors such as COVID-19 and floods can cause psychological stress responses in adolescents, and differences in coping styles can cause a range of behaviors in adolescents. Coping style is a significant factor leading to smartphone addiction among adolescents. Problem-focused coping strategies directly target the source of stress, prompting individuals to use positive coping styles to deal with the adverse consequences of the pandemic. Conversely, avoidance, denial, and fantasy employed as coping styles in dealing with stress are potentially strong risk factors for smartphone addiction (Stahl and Caligiuri, 2005; Duan et al., 2021). Therefore, adolescents with low levels of psychological resilience may experience tension, anxiety, depression, and other emotions in the face of emergencies or stressors. Thus, adolescents with low levels of psychological resilience may deal with stressors in harmful ways or by trying to escape from reality. Finally, poor coping styles or avoidant thoughts may also raise the risk of phone addiction.

### Limitations

This study had several limitations. First, using a convenience sample limits the generalizability of our results. Due to the cross-sectional study design, we could not produce longitudinal data. Therefore, we could not accurately deduce causal relationships between variables. Factors such as family environment, personality traits, peer relationships, and sleep quality may also affect mobile phone addiction among adolescents. Therefore, future studies should examine whether the relationship between Chinese adolescent resilience, coping style, DASS, and mobile phone addiction will change over time.

## Conclusion

This study explored the impact of resilience on mobile phone addiction among Chinese adolescents during a pandemic and flood. A structural equation model was utilized to synchronously examine the individual and continuous mediating roles of coping styles and DASS. The results suggest that a negative relationship exists between resilience and mobile phone addiction in this population. In addition, stress, anxiety, depression, and coping style significantly influence the risk of adolescent mobile addiction and play intermediary roles in Chinese adolescent resilience and mobile phone addiction. These results indicate the importance of mobile phone addiction and resilience for adolescents. The findings may also help educators and medical personnel distinguish between predictive factors for adolescent mobile phone addiction. They could be used to design effective interventions to treat and prevent mobile phone addiction in adolescents when facing future challenging or traumatic events.

## Data Availability Statement

The original contributions presented in this study are included in the article/supplementary material, further inquiries can be directed to the corresponding author.

## Ethics Statement

All procedures performed in studies involving human participants were approved by the Ethics Committee of Xinxiang Medical University (#XYLL-2018015). Written informed consent to participate in this study was provided by the participants’ or their legal guardian/next of kin.

## Author Contributions

HC designed the study. XL and SZ collected the data. AM analyzed the data and wrote the manuscript. YY and SG revised the manuscript. All authors contributed to the article and approved the submitted version.

## Conflict of Interest

The authors declare that the research was conducted in the absence of any commercial or financial relationships that could be construed as a potential conflict of interest.

## Publisher’s Note

All claims expressed in this article are solely those of the authors and do not necessarily represent those of their affiliated organizations, or those of the publisher, the editors and the reviewers. Any product that may be evaluated in this article, or claim that may be made by its manufacturer, is not guaranteed or endorsed by the publisher.
